# Effect of deep brain stimulation compared with drug therapy alone on the progression of Parkinson’s disease

**DOI:** 10.3389/fnins.2023.1330752

**Published:** 2024-01-08

**Authors:** Wenwen Dong, Chang Qiu, Yue Lu, Bei Luo, Xu Jiang, Lei Chang, Jiuqi Yan, Jian Sun, Weiguo Liu, Li Zhang, Wenbin Zhang

**Affiliations:** ^1^Department of Functional Neurosurgery, The Affiliated Brain Hospital of Nanjing Medical University, Nanjing, China; ^2^Department of Geriatric Medicine, The Affiliated Brain Hospital of Nanjing Medical University, Nanjing, China; ^3^Department of Neurology, The Affiliated Brain Hospital of Nanjing Medical University, Nanjing, China

**Keywords:** deep brain stimulation, disease progression, Parkinson’s disease, follow-up, UPDRS-III

## Abstract

**Background:**

Parkinson’s disease (PD) symptoms deteriorate with disease progression. Although deep brain stimulation (DBS) can effectively improve the motor signs of PD patients, it is not yet known whether DBS surgery, which is an invasive treatment modality, may change the progression of PD.

**Objective:**

The aim of this work was to compare the effect of DBS with that of drug treatment on the progression of PD.

**Methods:**

A total of 77 patients with PD with the Hoehn and Yahr scale (HY) stage of 2.5 or 3 were included, and were divided into 34 in the drug therapy alone group (Drug-G) and 43 in the DBS therapy group (DBS-G). All patients were subjected to a follow-up of 2 years, and disease severity was assessed by the Unified Parkinson’s Disease Rating Scale part III (UPDRS-III), the Montreal Cognitive Assessment (MOCA), the Hamilton Anxiety Scale (HAMA), and the Hamilton Depression Scale (HAMD) scores. In addition, the quality of life of patients and the burden on their family were assessed by the 39-item PD questionnaire (PDQ-39) scores, daily levodopa equivalent dose (LED), patient’s annual treatment-related costs, and the Zarit Caregiver Burden Scale (ZCBS) score. The changes in relevant scale scores between the two groups were compared at each follow-up stage.

**Results:**

The UPDRS-III score of the patients in the “off” state increased from year to year in both groups, and the degree of increase of this score was greater in the DBS-G than in the Drug-G group. The MOCA score in both groups began to decline in the 2nd year of follow-up, and the decline was greater in the Drug-G than in the DBS-G group. DBS treatment did not affect patients’ psychiatric disorders. The PDQ39, LED, costs, and ZCBS were negatively correlated with the follow-up time in patients in the DBS-G group, and positively correlated with the follow-up time in patients in the Drug-G.

**Conclusion:**

PD is progressive regardless of treatment. The findings from this follow-up study suggest that the disease progression of patients in DBS-G may be slightly faster compared to the drug-G, but the advantages of DBS are also evident. Indeed, DBS better improves patient’s motor signs and quality of life and reduces the family burden. In addition, DBS has less impact on patients in terms of cognitive and mental effects.

## 1 Introduction

Parkinson’s disease (PD) is a disabling chronic neurodegenerative disorder in which disease severity increases with disease duration (Cabreira and Massano, 2019). PD patients gradually develop motor signs such as bradykinesia, resting tremor or muscle rigidity, as well as non-motor signs such as olfactory loss, psychiatric symptoms or cognitive deficits due to the progressive apoptosis and necrosis of substantia nigra dopaminergic neurons, all having a serious impact on the patient’s quality of life (Schrag et al., 2000; Tolosa et al., 2021). Patients are treated with levodopa-based drugs in the early stages of the disease, which are effective in improving motor signs. However, the amount of oral medication and the type of medication the patient takes gradually increases as the disease progresses. Thus, long-term oral medications lead to complications such as anisocoria, motor fluctuations, and gastrointestinal reactions, which further reduce their quality of life (Schapira et al., 2013; Verschuur et al., 2019). Therefore, more and more patients and their families are more interested in deep brain stimulation (DBS) treatment.

DBS was first used by Prof. Benabid’s team in 1987 in clinical studies to control tremor in PD, and has been known ever since (Benabid et al., 1987). DBS has rapidly developed over the past 30 years because is minimally invasive, reversible, and modifiable. The principle of action of DBS is mainly to inhibit the abnormal neuronal electrical activity in the basal ganglia region of PD patients through electrical stimulation (Garcia et al., 2003; Brown et al., 2004; Meissner et al., 2005). DBS is effective in PD, but it is not yet known whether it influences peri-electrode neuronal cells. Some studies suggest that DBS is a double-edged sword. Although it affects the pathological neural activity, it also affects the normal physiological neural activity, leading to the deterioration of the partial motor function (Chen et al., 2006; Ray et al., 2009). Studies on the effects of DBS on neurons are usually observed in animal experiments, but the results are widely divergent. Some researchers believe that DBS has a neuroprotective effect (Jakobs et al., 2020; Knorr et al., 2022), others believe the opposite (Fischer et al., 2015, 2017), and they found that STN DBS does not protect the nigrostriatal system. Therefore, some PD patients remain concerned on the potential adverse effects of DBS.

In this study, patients who underwent DBS neurosurgical procedure were compared with patients treated with medication alone, and the relevant scores in the patients’ “off” state were used as a surrogate marker of disease severity (Menza et al., 1990; Mahlknecht et al., 2022) to evaluate the effects and advantages of DBS on PD progression.

## 2 Materials and methods

This study was approved by the Human Subjects Ethics Committee of the Affiliated Brain Hospital of Nanjing Medical University. Written informed consent was obtained from all participants. The work described has been carried out in accordance with The Code of Ethics of the World Medical Association.

### 2.1 Patient demographics and data acquisition

A total of 132 patients with primary PD attending the Department of Functional Neurosurgery and Department of Neurology at the Brain Hospital of Nanjing Medical University were recruited from June 2018 to June 2022, and 77 patients were finally enrolled due to loss of follow-up and data quality of the rest of the patients. The enrolled patients were divided into DBS therapy group (DBS-G) with 43 patients treated with DBS surgery, and drug therapy alone group (Drug-G) with 34 patients treated with medication alone. The inclusion criteria were as follows: (1) a definitive diagnosis of idiopathic PD; (2) patients with the Hoehn and Yahr scale (HY) stage 2.5 or 3 (Goetz et al., 2004); (3) no severe cognitive impairment or psychiatric disorders; (4) no medically coexisting disorders that could interfere with surgery or survival; (5) willingness and ability to undergo follow-up.

Disease severity was assessed by the Unified Parkinson’s Disease Rating Scale part III (UPDRS-III) score, the Montreal Cognitive Assessment (MOCA) score, the Hamilton Anxiety Scale (HAMA) and the Hamilton Depression Scale (HAMD) scores. Patients’ quality of life and family burden were assessed by the 39-item Parkinson’s Disease Questionnaire (PDQ-39) score, daily levodopa equivalent dose (LED) (Tomlinson et al., 2010), patient’s annual treatment-related costs, and the Zarit Caregiver Burden Scale (ZCBS) score (Zarit et al., 1980). All the above assessments were performed by the same neurologist.

The state of PD patients 72 h after discontinuing oral dopa agonists and 12 h after discontinuing oral levodopa preparations was considered as “OFF MED”; the state of patients 1 h after oral drug administration was considered as “ON MED”; the state of patients 40 min after turning off the implantable pulse generator (IPG) was considered as “OFF STIM”; the state of patients 30 min after turning on the IPG was considered as “ON STIM.”

The UPDRS III, MOCA, HAMA, HAMD, PDQ-39, LED, costs, and ZCBS scores of the medication–off status of the Drug-G patients were collected at the time of their enrollment, and at the 1st and 2nd year of follow-up. Additional assessment of the UPDRS-III scores of patients’ medication open status was performed at the second year of follow-up.

The 43 patients in the DBS-G group met the criteria for surgery and underwent DBS. The UPDRS-III, MOCA, HAMA, HAMD, PDQ-39, LED, annual treatment-related costs, and ZCBS scores of the patient’s OFF MED and OFF STIM status were collected at the time of their enrollment, and at the 1st and 2nd year of follow-up. Additional evaluation of the UPDRS-III scores for ON MED and ON STIM status was performed at the second year of follow-up.

### 2.2 Surgical procedure and postoperative management

The patients received general anesthesia throughout the DBS procedure. Electrode (model L301, PINS, Peking, China) implantation was performed using the leksell stereotactic system (Elakta, Stockholm, Sweden), and the electrodes were implanted in the subthalamic nucleus. The depth of anesthesia was controlled according to the bispectral index monitoring to facilitate the observation of potential changes in neuronal action during the operation to determine the depth of electrode implantation (Kwon et al., 2016). The postoperative computed tomography image of the patients was scanned and fused with the preoperative magnetic resonance to reconfirm that the electrodes were implanted in the subthalamic nucleus.

The patient’s IPG was turned on 1 month after surgery, and the electrode parameters were gradually increased using a titration approach while decreasing the patient’s drug dose. The patient’s motor signs were usually stabilized after 4–6 months. The adjustment of the patient’s programmed parameter and drug dose was performed by a neurologist with over 20 years of experience in PD assessment.

### 2.3 Statistical analysis

Statistical analysis was performed using GraphPad Prism 9 (GraphPad Software, San Diego, California, United States). Descriptive statistics of continuous variables and categorical variables were reported as mean ± standard deviation (SD) and as counts and percentages, respectively. Data with a normal distribution were expressed as mean ± standard deviation (x ± SD), while data without normal distribution were expressed as median (25th–75th percentile). Two-sample *t*-test or ANOVA were used for comparisons of groups that satisfied normal distribution, while Kruskal-Wallis test was used for comparisons of groups that did not satisfy normal distribution. The ANOVA started with a homogeneity test of variance. One way ANOVA was used for the comparison among groups with homogeneous variance, and welch ANOVA was used for comparison among groups with heterogeneous variance. The chi-square test was used for categorical data. A *p*-value < 0.05 was considered statistically significant.

## 3 Results

### 3.1 Demographic and clinical characteristics of the subjects at enrollment

Table 1 shows the comparison of the clinical characteristics of the patients in the Drug-G and DBS-G group in the OFF MED state at the time of the enrollment, and the results revealed no statistical difference between the two groups in terms of sex distribution, duration of the disease, HY stage distribution, and UPDRS-III. However, a statistically significant difference was found between the two groups in terms of age, MOCA, HAMA, HAMD, PDQ-39, annual treatment-related costs, and the ZCBS scores. A higher MOCA score indicates better cognitive function, while a higher UPDRS III score signifies poorer motor function. Additionally, higher scores in HAMA and HAMD reflect poorer mental status, and a higher PDQ39 score indicates a lower quality of life. Furthermore, a higher LED signifies a higher oral medication dosage, and elevated ZCBS scores indicate a heavier burden on caregivers. From Table 1, we can observe that Patients in the Drug-G are older than those in the DBS-G, and their cognitive level and mental status are slightly inferior to those in the DBS-G. Interestingly, although patients in the DBS-G took fewer oral medications per day, they had a poorer quality of life, and their family burden was higher than that of the Drug-G.

**Table 1:** Demographic and clinical characteristics of the subjects at enrollment.

Features	Drug-G	DBS-G	*p*-value
Gender, n (males/females)	15/19	21/22	0.82
Age, years	66 ± 8.06	56.88 ± 7.46	<0.001
Disease duration, years	6 (5, 8)	6 (5, 8)	0.79
H-Y grades, *n* (2.5/3.0)	12/22	18/25	0.64
UPDRS-III (OFF MED)	36 (32.75, 50)	42 (35, 48)	0.18
MOCA	27 (26, 29)	29 (28, 30)	0.004
HAMA	10 (6, 12)	4 (3, 7)	<0.001
HAMD	10 (6.75, 13.25)	5 (3, 5)	<0.001
PDQ-39	41.65 ± 13.56	48.21 ± 9.37	0.02
LED (mg)	579.43 ± 86.45	556.45 ± 90.49	0.26
Costs (¥)	9,500 (8,000, 12,000)	12,000 (10,000, 13,000)	0.001
ZCBS	17.88 ± 4.59	22.28 ± 5.83	<0.001

Data are presented as mean ± SD or median (25th–75th percentile). UPDRS-III, the Unified Parkinson’s Disease Rating Scale part III; MOCA, Montreal Cognitive Assessment; HAMA, Hamilton Anxiety Scale; HAMD, Hamilton Depression Scale; PDQ-39, the 39-item Parkinson’s Disease Questionnaire; LED, daily levodopa equivalent dose; costs, patient’s annual treatment-related costs; ZCBS, the Zarit Caregiver Burden Scale.

The chi-square test was used for between-group comparisons of categorical variables. For intergroup comparisons of quantitative data, the normal distribution test and then the chi-square test were performed. Mann Whitney’s test was used for not satisfying normal distribution, group *t*-test was used for satisfying normal distribution with equal variance, and Welch’s *t*-test was used for satisfying normal distribution with unequal variance.

### 3.2 Follow-up of the clinical characteristics of patients in the Drug-G group

Table 2 shows the comparison of the clinical characteristics of the patients in the Drug-G group at each follow-up time period, and the results revealed a statistically significant difference in the UPDRS-III, HAMA, HAMD, LED, annual treatment-related costs, and the ZCBS between the baseline-OFF MED and 1 year-OFF MED. In addition, a statistically significant difference was found in the UPDRS-III, MOCA, costs, and the ZCBS between 1 year-OFF MED and 2 year-OFF MED. HAMA, HAMD, LEDD, cost, and the ZCBS of the baseline-OFF MED were statistically different compared to the 2 year-OFF MED. Patients in the OFF MED Drug-G group had worsening motor symptoms each year. Their cognitive decline occurred in the second year of follow-up, the mental status was better than at the baseline, the quality of life declined in the first year, and the oral medications and family burden were gradually increased.

**Table 2:** Clinical characteristics of Drug-G patients.

Features	Drug-G			
	Baseline-OFF MED	1 year-OFF MED	2 years-OFF MED	2 year-ON MED
UPDRS-III	36 (32.75, 50)*#	37.5 (33.75, 50)^	39 (35, 52.25)	25.62 ± 8.09
MOCA	27 (26, 29)#	27 (26, 28.25)^	25.85 ± 2.69	–
HAMA	10 (6, 12)*#	8 (5, 9)	7 (4, 8)	–
HAMD	10 (6.75, 13.25)*#	8 (5, 11)	6.5 (3, 10)	–
PDQ-39	41.65 ± 13.56 #	43.09 ± 13.47	48.26 ± 11.34	–
LED (mg)	550 (533.4, 633.4)	612.5 (565.9, 737.55)	700 (566.7, 750)	–
Costs (¥)	9,500 (8,000, 12,000)*#	11000 (9000, 15000)	15000 (10000, 16000)	–
ZCBS	17.88 ± 4.59*#	19.91 ± 3.89	21.35 ± 3.88	–

For comparisons between multiple groups, the normal distribution test was performed first, and nonparametric test was used for those that did not satisfy the normal distribution, and then ANOVA was performed after satisfying the normal distribution; ordinary ANOVA test was used for equal variances, and Brown-Forsythe and Welch ANOVA were used for unequal variances.

*p < 0.05 baseline-OFF MED VS 1 year-OFF MED.

#*p* < 0.05 baseline-OFF MED VS 2 years-OFF MED.

^*p* < 0.05 1 year-OFF MED VS 2 years-OFF MED.

### 3.3 Follow-up of the clinical characteristics of patients in the DBS-G group

Table 3 shows the comparison of the clinical symptoms of patients in the DBS-G group who were subjected to a follow-up for 2 years, and the results revealed a statistically significant difference in the UPDRS III, PDQ-39, LEDs, costs and the ZCBS score between the baseline-OFF MED and 1 year-OFF MED, OFF STIM; A statistically significant difference was found in UPDRS-III and MOCA between 1 year-OFF MED, OFF STIM and 2 year-OFF MED, OFF STIM. A significant difference in the UPDRS-III, MOCA, PDQ-39, LEDD, costs, and the ZCBS score was found between the baseline-OFF MED and 2 year-OFF MED, OFF STIM. Patients in the DBS-G had a similar yearly worsening of motor symptoms during OFF MED and OFF STIM. Their cognitive level was also declining in the second year of the follow-up, the mental status was not significantly different across the follow-up phases, patients’ quality of life improved after surgery, oral medications were decreasing, and family burden was decreasing.

**Table 3:** Clinical characteristics of DBS-G patients.

Features	DBS-G			
	baseline-OFF MED	1 year-OFF MED, OFF STM	2 years-OFF MED, OFF STM	2 years-ON MED, ON STM
UPDRS-III	42 (35, 48)*#	44 (39, 49)^	48 (43, 53)	24 (5.15)
MOCA	29 (28, 30)#	28 (28, 30)^	28 (27, 30)	–
HAMA	4 (3, 7)	5 (3, 6)	5 (3, 7)	–
HAMD	5 (3, 5)	4 (3, 5)	4 (3, 6)	–
PDQ-39	48 (42, 54)	20 (14, 28)	22 (17, 32)	–
LED (mg)	556.45 ± 90.49*#	284.88 ± 72.81	290.12 ± 75.82	–
Cost (¥)	12,000 (10,000, 13,000)*#	5,000 (4,000, 6,000)	5,000 (4,000, 5,000)	–
ZCBS	22.28 ± 5.83*#	14.7 ± 4.03	15.16 ± 4.08	–

For comparisons between multiple groups, the normal distribution test was performed first, and nonparametric test was used for those that did not satisfy the normal distribution, and then ANOVA was performed after satisfying the normal distribution; ordinary ANOVA test was used for equal variances, and Brown-Forsythe and Welch ANOVA were used for unequal variances.

*p < 0.05 baseline-OFF MED VS 1 year-OFF MED, OFF STM.

#*p* < 0.05 baseline-OFF MED VS 2 years-OFF MED, OFF STM.

^*p* < 0.05 1 year-OFF MED, OFF STM VS 2 years-OFF MED, OFF STM.

### 3.4 Effect of drugs and DBS on the progression of PD

**Figure 1:**
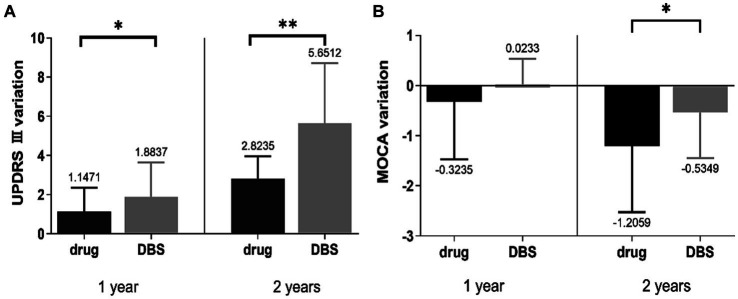
**(A)** Suggested that the patients in the DBS-G had a greater degree of progression in motor function than the Drug-G in both the first and second years of follow-up, **(B)** showed that the MOCA scores of the Drug-G declined more than those of the DBS-G in the second year. *p < 0.05; **p < 0.01.

**Figure 2:**
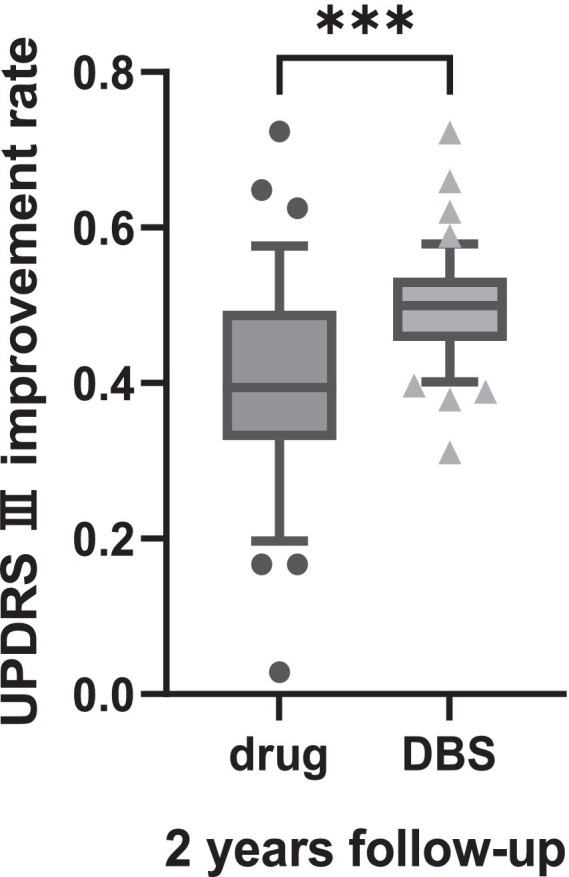
Suggested that during the second year of follow-up in the “ON” state the degree of improvement in motor function was significantly greater in the DBS-G patients than in the Drug-G. ***p < 0.001.

The change in the UPDRS-III and MOCA scores in Tables 2, 3 revealed that motor signs and cognition were progressing in both groups. The degree of change in UPDRS III and MOCA scores was compared between the two groups, and the results suggested that the patients in the DBS-G group had a greater degree of reduction of motor function than the Drug-G in both the first and second year of the follow-up (Figure 1A). However, the MOCA scores of the Drug-G group declined more than those in the DBS-G group in the second year (Figure 1B). Then, the rate of improvement in the UPDRS-III in the “on” state was compared between the two groups after two years of follow-up. The improvement rate refers to the difference between the patient’s UPDRS III score in the “off” and “on” states in the second year, and then dividing by the UPDRS III score in the “of” state in the second year. The results suggested that the degree of improvement in motor function was significantly greater in the DBS-G patients than in the Drug-G (Figure 2).

**Figure 3:**
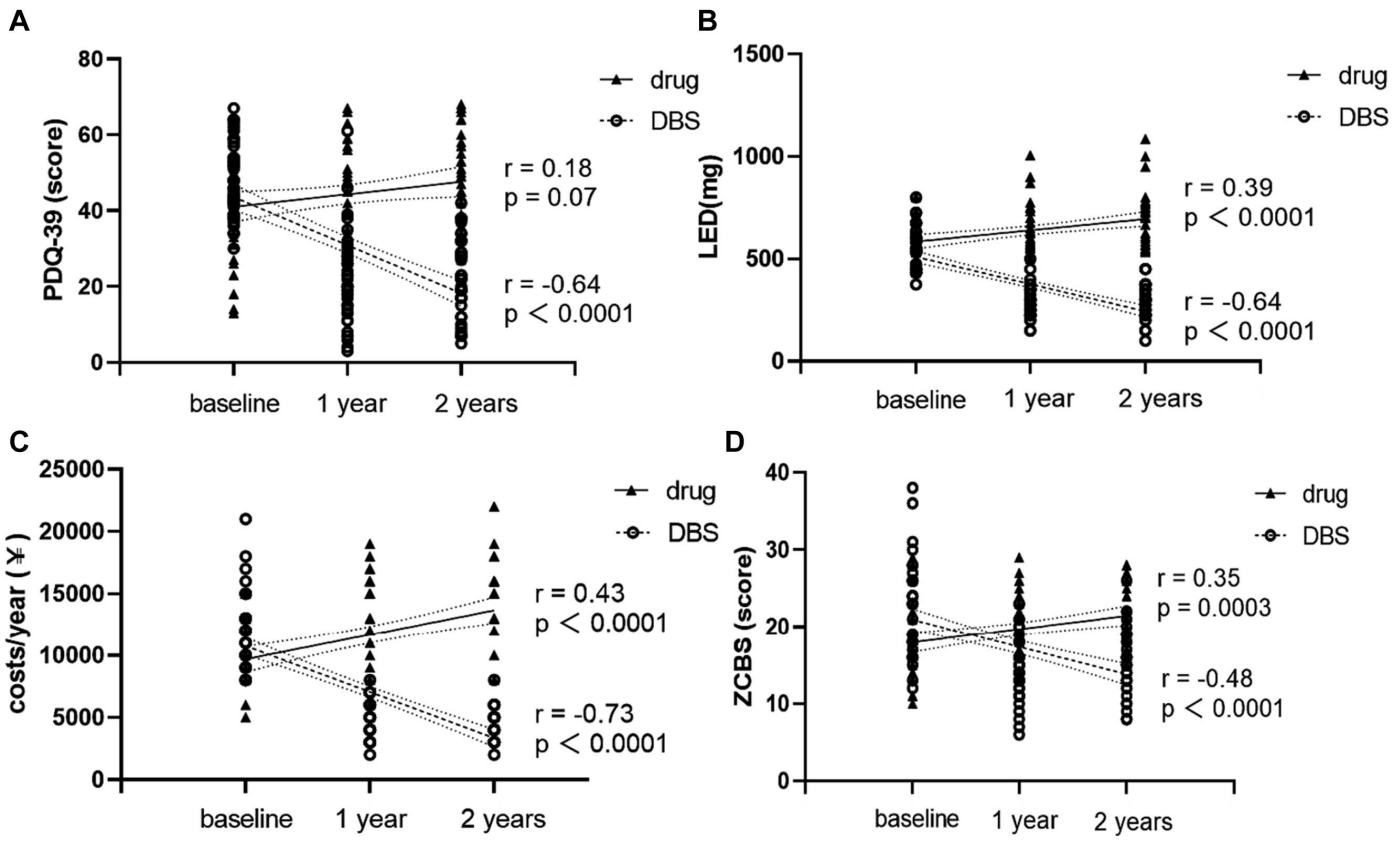
**(A)** shows the changing trends of PDQ-39 for two groups of patients over time. **(B)** shows the changing trends of LED for two groups of patients over time. **(C)** shows the changing trends of the cost for two groups of patients over time. **(D)** shows the changing trends of the ZCBS scores for two groups of patients over time. Nonparametric spearman analysis was used for correlation analysis.

Next, the correlation of PDQ-39, LED, costs, and the ZCBS with the follow-up time in the two groups of patients was analyzed. The results in Figure 3 show that PDQ39, LED, costs, and the ZCBS score of patients in the DBS group were negatively correlated with the follow-up time, while LED, cost, and ZCBS of the Drug-G group were positively correlated with the follow-up time. These results indicated that the DBS-G significantly improved the quality of life of the patients compared with the Drug-G, the patients’ oral medication and the family burden were reduced.

## 4 Discussion

The pathology of PD is characterized by the degenerative necrosis of dopamine neurons in the basal ganglia region. The patient’s symptoms worsen with the duration of the disease. To date, no effective means available to cure the disease or slow its progression (Fox et al., 2018; Vijiaratnam et al., 2021). PD is becoming more and more well known with the development of medical care. Early detection, early diagnosis and early treatment are considered important measures for the slowing down and treatment of PD. The diagnosis of PD is usually followed by the treatment with anti-PD medications. After the “honeymoon effect” of the medications, the patient may experience some side effects that reduce the quality of life. Some studies found that dopaminergic therapies are effective in controlling motor symptoms, but no evidence is available indicating that the drugs alter disease progression or normalize life expectancy (Macleod et al., 2014; Verschuur et al., 2019; Cilia et al., 2020; Vijiaratnam et al., 2021). In addition, levodopa accelerates the loss of substantia nigra dopaminergic neurons endings or alters the function of dopamine transporters (Armstrong and Okun, 2020).

Patients consider DBS surgery when motor complications arise, but the invasiveness compared to medication still leaves patients with concerns. The results of this study revealed that the UPDRS-III score increased year after year in both groups (drug alone and DBS treatment), indicating that the patients’ motor symptoms were worsening. Currently, an increasing number of researchers are focusing on the importance of minimal clinically important differences (MCID). MCID refers to the level of change in the effect of treatment or intervention that is considered meaningful by patients or clinical professionals. Establishing MCID helps researchers and clinicians to assess whether the treatment effect is clinically significant, and aids in the design of clinical trials and interpretation of research results. Some researchers have proposed that a 5-point difference in UPDRS-III scores represents the MCID (Wu et al., 2019). Figure 2A show patients in the DBS-G group had a greater degree of increased UPDRS-III scores in the second year than those in the Drug-G group, suggesting that the disease progression of patients in DBS-G may be faster compared to the drug-G. It has been suggested that the insufficient discontinuation of medication can compromise the assessment of PD severity, and an accurate assessment of a patient’s original symptoms requires the discontinuation of medication for more than 2 weeks to ensure that the antiparkinsonian medication is fully metabolized in the body (Olanow et al., 1995; Hauser et al., 2000). Since PD patients are usually unable to be subjected to a 2-week discontinuation, a more common discontinuation, i.e., a 24-h discontinuation of all antiparkinsonian medications was chosen in this work except for the dopamine agonists, which were discontinued for 3 days. In addition, since the daily oral levodopa dose in the Drug-G group was significantly higher than that in the DBS-G group, the residual concentration of the drug in the patients’ bodies might be different although the discontinuation time was the same, which might be one of the reasons for the bias of the results. However, the 2 years follow-up revealed that the improvement rate of UPDRS-III in the DBS-G group was significantly higher than that in the Drug-G group, suggesting that the efficacy of DBS-G was superior to that of Drug-G.

As regards the non-motor symptoms, patients in both two groups showed a reduction in MOCA scores in the second year of follow-up, with patients in the Drug-G having a greater reduction in MOCA scores than those in the DBS-G. Some researchers believe that the MCID for MOCA is 2.15 (Krishnan et al., 2017). Therefore, despite the statistically significant difference in the decrease in MOCA scores between the two groups over a 2-year period, considering the MCID, we believe that the slight changes in MOCA may not have clinical significance, and the majority of participants’ MOCA scores are still considered normal. To date, the results regarding the effect of DBS on the cognitive level of patients are still divided; some studies believe that DBS aggravates the cognitive level of patients, and some others believe that cognitive decompensation is related to the age of the patients. Based on the results of the present study, we speculate that the decline in the cognitive level of the patients might be related to the aging or to the type and dose of the oral medications. The type and dose of medication of the patients are significantly reduced after DBS surgery; thus, the cognitive alteration might be related to the oral medications as well. Studies related to mental disorders demonstrated that approximately 20 to 30% of patients with PD have symptoms of depression, and 20–52% of patients have symptoms of anxiety (Schrag et al., 2002; Schrag, 2004; Broen et al., 2016) and mental disorder, which are also affected by the decline in the quality of life of the patients. The present study included patients with mild symptoms of mental disorders, and no statistically significant difference was found in the scores of psychiatric symptoms in the DBS-G group in each follow-up phase; thus, our conclusion was that DBS did not change the psychiatric state of the patients.

Patients’ ability to perform activities of PDQ-39 scores increased in the Drug-G group, while the scores decreased in the DBS-G group, indicating that DBS improved patients’ quality of life. In addition, the amount of medication in the Drug-G group gradually increased, while that in the DBS group gradually decreased. Moreover, our team believed the importance of focusing not only on PD patients, but also focus on their family burden; thus, this factor was assessed through the economic expenditure and the ZCBS scores. The results showed that the DBS-G patients’ treatment expenditure and the ZCBS scores decreased year by year, whereas those of the drug-G have been increasing year by year. Therefore, our hypothesis was that the DBS improved the patient’s motor signs and enhanced the patient’s ability to live on their own, thus improving the stress of the caregivers.

## 5 Strengths and limitations

### 5.1 Strengths

Relatively few studies have been performed on the impact of DBS on disease progression in patients with PD. Since patients may consider DBS surgery when disease progression reaches approximately an intermediate stage, this study included patients with HY stage 2.5 and 3. Moreover, medication alone and DBS treatment were followed and compared over time, to evaluate not only the motor and non-motor signs, but also the family burden of PD patients, which is usually ignored.

### 5.2 Limitations

The number of patients included in this study was relatively small and the follow-up period was not long. Thus, it is necessary to conduct ongoing follow-up on these participants. Another issue is the effect of off-drug time on the natural disease manifestations of the patients, as the drugs take time to be completely metabolized. Lastly, we may need to consider the concept of minimum clinically important difference, which may hold value in assessing the clinical significance of the magnitude of the observed changes.

## 6 Conclusion

Overall, PD is still progressive regardless of the treatment. The findings from this follow-up study suggest that the disease progression of patients in DBS-G may be slightly faster compared to the drug-G, but the advantages of DBS are evident. Indeed, it not only improves the motor signs of the patients, but also significantly improves the quality of life of the patients and reduces the burden on the patients’ families. In addition, DBS may be smaller than drugs in terms of cognitive and psychiatric disorders effects.

Currently, new treatment concepts, such as disease-modifying therapy, gene therapy, and stem cell therapy, are still in the research phase. The treatment of PD is still based on medication. Some studies found that the time to motor signs is not correlated with the time of pharmacologic intervention, but rather with the amount of medication and the duration of the disease (Cilia et al., 2014). Combined with the results of this study, our suggestion is that patients should receive medication in the early stages of the disease, and then they can choose DBS therapy when the dose of medication gradually increased or when there are fluctuations in the motor signs. At the end of this article, it is our hope that this research will generate increased attention from the research community towards examining the effects of different therapeutic approaches on the progression of Parkinson’s disease. By doing so, we aspire to offer a more comprehensive theoretical foundation to inform clinical practitioners in their decision-making processes.

## Data availability statement

The original contributions presented in the study are included in the article/supplementary material, further inquiries can be directed to the corresponding author.

## Ethics statement

The studies involving humans were approved by the Human Subjects Ethics Committee of the Affiliated Brain Hospital of Nanjing Medical University. The studies were conducted in accordance with the local legislation and institutional requirements. The participants provided their written informed consent to participate in this study. Written informed consent was obtained from the individual(s) for the publication of any potentially identifiable images or data included in this article.

## Author contributions

WD: Data curation, Formal Analysis, Methodology, Writing – original draft. CQ: Data curation, Investigation, Methodology, Writing – original draft. YL: Data curation, Software, Supervision, Writing – original draft. BL: Investigation, Software, Writing – review & editing. XJ: Data curation, Formal Analysis, Investigation, Writing – original draft. LC: Data curation, Formal Analysis, Writing – original draft. JY: Formal Analysis, Writing – review & editing. JS: Data curation, Investigation, Writing – review & editing. WL: Conceptualization, Investigation, Writing – review & editing. LZ: Validation, Writing – review & editing. WZ: Conceptualization, Funding acquisition, Resources, Writing – review & editing.
